# *Coxiella*-Like Endosymbiont of *Rhipicephalus sanguineus* Is Required for Physiological Processes During Ontogeny

**DOI:** 10.3389/fmicb.2020.00493

**Published:** 2020-04-22

**Authors:** Michael Ben-Yosef, Asael Rot, Mustafa Mahagna, Einat Kapri, Adi Behar, Yuval Gottlieb

**Affiliations:** ^1^Koret School of Veterinary Medicine, The Robert H. Smith Faculty of Agriculture, Food and Environment, The Hebrew University of Jerusalem, Rehovot, Israel; ^2^Kimron Veterinary Institute, Bet Dagan, Israel

**Keywords:** ticks, arthropod symbiosis, hematophagy, antibiotic treatment, reproductive fitness

## Abstract

Obligatory hematophagous arthropods such as lice, bugs, flies, and ticks harbor bacterial endosymbionts that are expected to complement missing essential nutrients in their diet. Genomic and some experimental evidence support this expectation. Hard ticks (Acari: Ixodidae) are associated with several lineages of bacterial symbionts, and very few were experimentally shown to be essential to some aspects of tick’s fitness. In order to pinpoint the nature of interactions between hard ticks and their symbionts, we tested the effect of massive elimination of *Coxiella*-like endosymbionts (CLE) by antibiotics on the development and fitness of the brown dog tick (*Rhipicephalus sanguineus*). Administration of ofloxacin to engorged (blood fed) nymphs resulted in significant and acute reduction of their CLE loads – an effect that also persisted in subsequent life stages (aposymbiotic ticks). As a result, the post-feeding development of aposymbiotic female (but not male) nymphs was delayed. Additionally, aposymbiotic adult females needed a significantly prolonged feeding period in order to replete (detach from host), and had reduced engorgement weight and a lower capacity to produce eggs. Consequently, their fecundity and fertility were significantly reduced. Eggs produced by aposymbiotic females were free of CLE, and the resulting aposymbiotic larvae were unable to feed successfully. Our findings demonstrate that the observed fitness effects are due to CLE reduction and not due to antibiotic administration. Additionally, we suggest that the contribution of CLE is not mandatory for oocyte development and embryogenesis, but is required during feeding in females, when blood meal processing and tissue buildup are taking place. Presumably, under these extreme physiological demands, CLE contribute to *R. sanguineus* through supplementing essential micro- and macronutrients. Further nutrient complementary studies are required to support this hypothesis.

## Introduction

Arthropods that feed exclusively on restricted diets such as plant sap or vertebrate blood rely on microbial symbionts to supplement missing essential nutrients in their diet (Buchner, 1965). Such nutritional symbioses are ubiquitous in obligatory blood feeders like Tsetse flies and bedbugs, where bacterial symbionts synthesize B vitamins lacking in the blood meal (Hosokawa et al., 2010; Michalkova et al., 2014). These vitamins serve as essential cofactors for major enzymatic activities and are crucial for cell function and energy production (Depeint et al., 2006).

Ticks (Acari: Ixodida) are obligatory blood feeders and are suggested to be the earliest organisms to evolve blood-feeding capabilities (in the Devonian; Mans and Neitz, 2004). Various tick species studied to date harbor endosymbiotic bacteria which are assumed to supplement their hosts with B vitamins (Duron et al., 2017). These include *Coxiella*, *Francisella*, and *Rickettsia* species, as well as other bacteria. The most widespread of these are *Coxiella*-like endosymbionts (CLE), which show tight co-evolution patterns with hard ticks of the genus *Rhipicephalus* (Ixodidae; Duron et al., 2015; Tsementzi et al., 2018). All CLE genomes studied so far are reduced in size and retain some functional pathways for synthesizing B vitamins (Gottlieb et al., 2015; Smith et al., 2015; Guizzo et al., 2017; Tsementzi et al., 2018). This is also the case in studied genomes of tick-associated *Francisella*-like endosymbionts (Gerhart et al., 2016; Duron et al., 2018).

The specific host recognition behavior and prolonged feeding strategy of hard ticks constrain laboratory rearing procedures of most species, including *Rhipicephalus* species, to the use of live hosts. Artificial feeding systems, where ticks feed on drowned vertebrate blood, are difficult to maintain, and currently support successful rearing of only a few tick species (Kröber and Guerin, 2007; Krull et al., 2017). Consequently, empirically determining the involvement of symbionts in the biology and nutrition of hard ticks is limited due to technical difficulties. Nevertheless, some experimental evidence for the contribution of CLE to the fitness of hard ticks has been accumulated.

Suppression of the tick microbiome by antibiotics was first employed on *Amblyomma americanum*, where either tetracycline or rifampicin was injected to engorged nymphs and adult females. Treated ticks had reduced CLE loads, decreased weight of nymphs, and fewer and less viable offspring (Zhong et al., 2007). In *Haemaphysalis longicornis*, injection of tetracycline into adult ticks, or feeding their hosts with antibiotics, resulted in reduced loads of CLE, and had significant effects on female weight, feeding time, fecundity, oviposition period, and egg hatching (Zhang et al., 2017). In the cattle tick *Rhipicephalus microplus*, tetracycline treatment of either adult ticks or the vertebrate host had no effect on the fitness of adult females or on egg development, but reduced CLE loads in the offspring, which in turn could not complete their development beyond the metanymph stage (Guizzo et al., 2017).

Recently, the removal of *Francisella*-like endosymbionts from the soft tick *Ornithodoros moubata* (Argasidae) using rifampicin in an artificial feeding system, showed significant impact on life history traits including decrease in molting and adult emergence and weight, as well as physical abnormalities. These effects were reverted after feeding treated ticks with B vitamins (Duron et al., 2018). Taken together, tick endosymbiotic bacteria, including CLE are assumed to be essential for reproduction and development, presumably through supplementing essential nutrients missing in the blood meal.

The aim of this study was to determine the effect of CLE on development and fitness of the brown dog tick *Rhipicephalus sanguineus*. We hypothesized that elimination of CLE would compromise the tick’s ability to utilize the blood meal and eventually hamper development and reproduction. To test this assumption, we first screened for the most effective antibiotic agent against CLE, and later examined the consequences of symbiont elimination on nymph post-feeding development time and female reproductive success. Simultaneously, we characterized the microbiome associated with the tick and determined the abundance of CLE during the nymphal and adult life stages and according to treatment.

## Materials and Methods

### CLE Abundance and Suppression by Antibiotics

**FIGURE 1:**
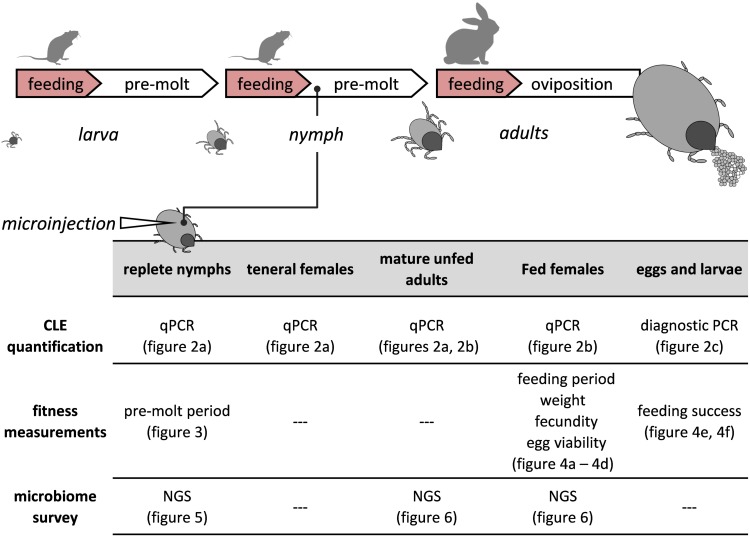
A visual depiction of the tick’s life cycle relative to the experimental procedures. Antibiotics or saline were injected once only to replete nymphs. Development and fitness measurements, symbiont quantifications (qPCR, PCR) and microbiome surveys (NGS) were performed on injected nymphs and subsequent life stages.

To obtain aposymbiotic ticks with reduced CLE loads, we microinjected engorged nymphs with saline containing antibiotics. Consequent effects on nymph development and the fitness of females developing from such nymphs were subsequently recorded (Figure 1). Antibiotics were selected based on their previously determined efficacies against the Q-fever agent *Coxiella burnetii* (Yeaman et al., 1987; Raoult et al., 1991) as well as their suppressive effect on CLE in other ticks (Zhong et al., 2007; Zhang et al., 2017). These studies highlighted ofloxacin, rifampicin, and tetracycline as suitable candidates having potent effects on *Coxiella* or *Coxiella*-like bacteria. All agents are broad-spectrum antibiotics that arrest bacterial replication by interfering with DNA, RNA, or protein synthesis, respectively.

Stock solutions were prepared in sterile saline (0.9% NaCl, pH 6.5; ofloxacin and tetracycline) or dimethyl sulfoxide (DMSO) diluted with sterile saline (1:5 v/v, respectively; rifampicin) and adjusted to a final concentration of 7 mg/ml. Tetracycline and rifampicin were dissolved directly in their corresponding solvents. Ofloxacin was solubilized through acidification with hydrochloric acid (1:700 v/v saline:HCl, respectively, pH 6.0). Sterility was insured by preparing and handling solutions in a laminar flow hood. All materials were obtained from Sigma, Israel.

Microinjections were performed on replete nymphs under a dissecting microscope using a 5-μl syringe (Hamilton, Reno, NV) coupled to a glass needle (pulled on a Sutter P-87 machine) and a microprocessor-controlled injection module (UMP3/SYS-Micro 4; WPI, United States). Injection volume was individually adjusted to deliver a dose of 50 ng of antibiotic for each milligram of body weight (or an equivalent volume of antibiotic-free solution). Prior to each injection, ticks were dorsally affixed to a microscopic slide and positioned perpendicularly to the needle. Ethanol (70%), applied by a delicate brush, was used to disinfect each tick as well as the needle prior to each injection. The needle tip was subsequently inserted adjacent to the coxa of the right foreleg, while pointing away from body center. A single injection volume was then discharged at a rate of 5 nl/s and the needle was left in place for additional 30 s before retracted to allow the solution to disperse in the hemocoel. None of the nymphs suffered significant injury during the procedure and all successfully recovered and continued their post-feeding development.

### Effect of CLE on Nymph Post-feeding Development

*Rhipicephalus sanguineus* nymphs and adults used in this study originated from the progeny cohort of a single female derived from a tick colony maintained at the Kimron Veterinary Institute research labs (Israel). Colony ticks are regularly reared on gerbils (*Meriones tristrami*) as larvae and nymphs, and on rabbits (*Oryctolagus cuniculus*) as adults.

To determine the effect of CLE on nymph development, fully engorged nymphs that detached from the host (collected during two, subsequent 24-h intervals) were weighed to the nearest 0.01 mg and randomly assigned to treatment groups of similar mean weight and variance. During the next 48 h, nymphs were microinjected with sterile saline solutions containing or excluding antibiotics (see above), individually distributed into cap-perforated tubes and maintained in a dark climate chamber (25 ± 1°C, 85 ± 5% RH) to complete their post-feeding development. During the subsequent weeks, we recorded the molting of nymphs every 24 h until all ecdysed as adult ticks. The sex of the resulting adults was recorded. These experiments were replicated twice.

**FIGURE 2:**
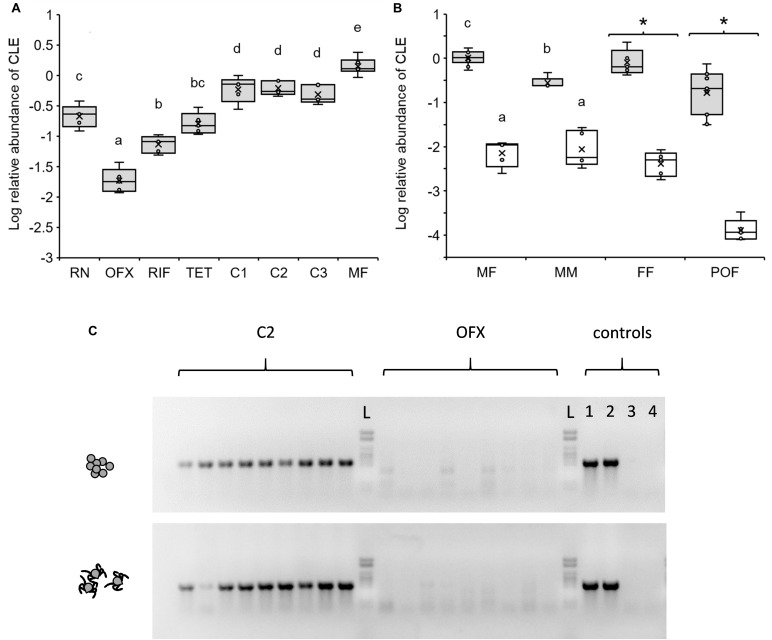
Relative abundance of CLE in different life stages and according to treatment as quantified by qPCR. **(A)** CLE abundance in replete nymphs (RN), teneral 3-day-old antibiotic-treated or non-treated females (see below for abbreviations) and mature 40-day-old females (MF) (*n* = 5–6 in each group). Antibiotics were administered in saline (ofloxacin, OFX or tetracycline, TET) or saline:DMSO solution (rifampicin, RIF), and implemented by injection to nymphs at 50 ng/mg weight. Control treatments included no treatment (C1) and saline (C2) or saline:DMSO (C3) injections. **(B)** CLE abundance in mature 40-day-old, unfed females (MF) and males (MM), feeding females (sampled 7 days after release on a host, FF), and post oviposition females (POF) previously injected as nymphs with ofloxacin (white boxes) or saline (shaded boxes). Different letters above means or asterisks (*), denote significant difference (Tukey HSD or ANOVA comparisons, *P* < 0.05; *n* = 5–11 in each group). **(C)** Diagnostic PCR detecting the 16S rRNA gene of CLE in 9 eggs (upper panel) and 9 larvae (lower panel) produced by females injected as nymphs with saline (C2) or ofloxacin (OFX). A 1kb DNA ladder (L), and two CLE positive (1,2) and negative (no template; 3,4) control assays are included in each panel. The tick’s 18S rRNA gene was consistently amplified and detected in all samples (not shown).

Our experiments indicated that different antibiotics varied in their potency against CLE (see section “Results,” Figure 2A) and suggested that nymph development time was differently affected by the antibiotics we tested (Supplementary Figure S1). Subsequent experiments proceeded using the most effective antibiotic against CLE (ofloxacin) and estimated the effects of CLE suppression and nymph weight and sex on nymph post-feeding development (time to adult ecdysis).

### Effect of CLE on Female Fitness

To determine the contribution of CLE to feeding and reproduction in adult females, we compared the time to repletion, engorgement weight, fecundity and egg viability of females reared on rabbits, and previously treated (or not) as nymphs with antibiotics (Figure 1). To achieve ca. 30 ticks in each experimental group, these experiments were replicated twice.

Unfed females injected with ofloxacin or saline solutions as nymphs, and non-treated males completed their pre-feeding maturation period in a dark climate chamber (20 ± 1°C, 85 ± 5% RH) during 10–11 weeks following ecdysis. Ticks were primed for feeding by providing a daily cycle of 16 h light:8 h dark, 27 ± 1°C and 85 ± 5% RH for 3–4 days, after which females of each treatment group (*n* = 17–30) were enclosed together with males (*n* = 14–21; 1:1.1–1.5 male:female ratio) in a cloth pouch sealed to the ears of a single female New-Zealand white rabbit (age: 16 weeks, average weight: 3.268 kg, min: 3.055, max: 3.450), similarly to procedures described by Levin and Schumacher (2016). During the next 21 days, tick attachment and feeding progress were monitored daily and engorged females leaving the host were collected and counted every 24 h. Ticks intended for qPCR (*n* = 5 in each group) were sampled 7 days after release on the rabbit, surface sterilized, and frozen (−80°C) until processed. Any ticks remaining on the rabbit were removed at the end of the experiment to confirm that all females and males were retrieved.

Replete females were weighed (to the nearest 0.01 mg) and individually sealed in cap-perforated 2-ml tubes placed horizontally in a climate chamber at 25 ± 1°C, 85 ± 5% RH and 16:8 L:D, where oviposition took place. At the end of oviposition (denoted when less than 3 eggs per day were produced), each female and egg mass were weighed, and females were additionally measured for body size (scutal index; Obenchain et al., 1980), surface sterilized, and preserved frozen until processed for qPCR. Eggs were allowed to develop and hatch for as long as 30 days after larvae were first sighted [long enough to confirm that all viable eggs had hatched (Heath, 1978)]. The content of each tube, including larvae and any remaining undeveloped eggs, was subsequently frozen (−20°C) in 95% ethanol. Hatching rate was determined for each egg mass by evenly spreading the content of each tube on a 90-mm Petri dish and determining the ratio of larvae to larvae + unhatched eggs in 3–6, randomly chosen, 10 × 14 mm fields using a stereomicroscope (Zeiss, Germany). Fields were digitally captured (Zeiss Axiocam) and counting was performed *in silico* using a software tool designed *ad hoc*.

### Effect of CLE on Larval Development

Finally, we determined the ability of larvae to successfully feed on a host. Larvae collected from hatched egg masses of antibiotic-treated (*n* = 6 egg masses) or saline-injected (*n* = 2) females were distributed into separate 1.5-ml tubes (1 or 3 tubes per female; ofloxacin-treated and saline-injected females, respectively; total of six tubes per treatment group) and weighed (7.6–28.6 mg larvae/tube). Each tube was subsequently opened and placed in the cage of a single gerbil to which larvae could attach and feed. During the following days, we collected and counted replete larvae leaving their hosts and finally compared these numbers between treatment groups, according to the initial amount (mg) of larvae released on the host. These experiments were supplemented by validating the presence or absence of CLE in eggs and larvae through diagnostic PCR (as described below).

### Identification and Quantification of CLE

Quantitative PCR was employed to examine the efficiency of antibiotic treatments and estimate CLE density *in vivo* at representative time points throughout the tick’s life cycle. Nymphs were examined 7 days post-injection, and adult, non-fed females were examined at 3 and 40 days after ecdysis [teneral (newly emerged) and mature, respectively], when semi-engorged, and at the end of oviposition (*n* = 5–10 in each group). Non-fed, mature males (*n* = 5 in each group) were examined 40 days post-ecdysis. Genomic DNA was extracted using the Qiagen blood and tissue kit (Hilden, Germany) according to the manufacturer’s instructions. Prior to DNA extraction, ticks were externally rinsed in a mild detergent solution (1% Alconox, United States) for 1 min, surface sterilized in 1% sodium hypochlorite (1 min), and finally washed three times in sterile saline. The CLE 16S and tick 18S rRNA gene copies were subsequently quantified by SYBR green-based qPCR on a SteponePlus machine (Applied Biosystems, CA, United States) using target-specific primers for detection of CLE in *Rhipicephalus turanicus*, as previously described (434f-1004r; Lalzar et al., 2012, 2014). *R. turanicus* is a member of the *R. sanguineus* species complex and both associate with closely related CLEs matching in their 16S rRNA gene sequence (99.9% nucleotide identity, Tsementzi et al., 2018). Quantitation of the tick’s rRNA gene was accomplished using a costume designed primer pair (673f-909r) designed using primer3^1^ and tested under blastn algorithm in NCBI. Readings were conducted in 20-μl triplicates containing 8 pmol of each primer and 30 ng of template DNA in ABsolute Blue qPCR mix (Thermo, MA, United States). 16S and 18S rRNA gene copy numbers (measured down to 180 and 1711 copies of the DNA target, respectively) were extrapolated using calibration curves generated from five 10-fold dilutions of standards (Plasmids containing the corresponding CLE 16S or tick 18S rRNA gene inserts). Melting curves were traced after each assay to confirm that fluorescence emanated from specific PCR products and amplicons resulting from antibiotic-treated and non-treated females (*n* = 11) were additionally sequenced and successfully matched to their original templates. PCR conditions, standard curve variables, and amplification data are provided in Supplementary Table S1.

Diagnostic PCR was used to examine the prevalence of CLE in eggs (14 days after oviposition; *n* = 3) and newly hatched larvae (*n* = 3) of antibiotic-treated or non-treated females (*n* = 6 in each group). Following surface sterilization and deactivation of DNA-cleaving enzymes [incubation in 10 μl of phosphate buffered saline (PBS, Biological Industries, Israel) at 95°C for 3 min], DNA was extracted from individual samples as previously described (Lalzar et al., 2012). PCR was performed on 5 μl of DNA extract in 25 μl of Gotaq G2 green master PCR mix (Promega, Wisconsin, MD, United States) using *Coxiella*-specific primers (as above) on a C1000 Thermal cycler (Bio-Rad laboratories, CA, United States). Products (10 μl) were electrophoresed on 1.5% agarose gel containing RedSafe nucleic acid stain (Intron biotechnology, South Korea) and digitally recorded on a UV transillumination table (EndoroGDS, Labnet International Inc., NJ, United States). To validate our DNA extraction procedure and confirm the signal of CLE, we similarly targeted the tick’s 18S rRNA gene in our samples using the 673f-909r primer-pair (as above). Plasmids containing CLE 16S or tick 18S rRNA gene inserts, and blank DNA extractions served as templates for positive and negative control reactions, respectively.

### Characterization of Nymph and Adult Tick Microbiomes

High-throughput sequencing was used to examine the diversity of bacteria associated with the different life stages of the tick and determined if deviations in community composition are stage dependent or resulted from treating ticks with antibiotics. DNA was extracted from antibiotic-treated and non-treated ticks: repleted nymphs and unfed and fed females as described above (*n* = 5 in each group). Additionally, we included unfed wild females (collected at Caesarea, Israel, April 2016; *n* = 5). The bacterial 16S rRNA gene diversity in each sample was subsequently analyzed at the DNA Services Facility of the University of Illinois, Chicago, United States. We employed the Illumina MiSeq platform (Illumina, United States) and Standard targeted-amplicon sequencing (Green et al., 2015) with the 515F-806R primer pair targeting the V4 region of the bacterial 16S rRNA gene (Caporaso et al., 2012).

Sequences were processed and analyzed in MOTHUR v.1.41.1 (Schloss et al., 2009) according to the standard MiSeq protocol^2^. Following the trimming of primer sequences, contigs containing no ambiguities and up to 8-base-long homopolymers were aligned to the SILVA v132 reference database (Quast et al., 2012) and filtered so that all overlapped and spanned 254 bp in length with no overhangs. Pre-clustering (Huse et al., 2010) was performed with up to 4-base-divergence among sequences, and chimeras were removed using MOTHUR’s implementation of UChime (Edgar et al., 2011). Finally, sequences of non-bacterial origin, resulting from chloroplast and mitochondria, or unclassified at the kingdom level were removed. Resulting sequences were grouped into Operational Taxonomic Units (OTUs) having 97% sequence similarities according to the current SILVA taxonomy (v132). Singletons and doubletons were removed from the datasets. Genus abundance data were not rarefied, following the recommendation of McMurdie and Holmes (2014). Resulting rarefaction curves are depicted in Supplementary Figure S2. Sequencing and diversity data after final processing (see below) are provided in Supplementary Table S2.

### Statistical Analyses

(a) Nymph development time: The effect of suppressing CLE (“treatment”) on nymph post-feeding development time (“days to ecdysis”) was examined by full factorial, three-way analysis of co-variance (ANCOVA) with nymph sex and replicate as additional fixed factors. Nymph weight was introduced as a co-variate in the model after confirming the correlation with development time by regression analysis (Supplementary Table S3). Within the model, means were separated by Tukey HSD or contrast *t*-test comparisons. The normal distribution and homogeneity of residuals was confirmed by Shapiro–Wilk and Bartlett’s tests, respectively.

Survival analysis was additionally employed to confirm the effect of “treatment” on development time (“days to ecdysis”). These analyses were performed on pooled data from both replicates and separately for males and females.

(b) Female fitness: We tested the effect of CLE on female engorgement weight, feeding period, fecundity (egg-mass weight), weight to eggs conversion ratio, and egg hatching rate. Prior to analyses, we tested the correlation between body size (scutal index) and weight (linear regression analysis), and females which failed to attain engorgement weights fitting within the data distribution of conspecifics were omitted (outliers, *n* = 2 from each treatment group). The effects of “body size,” CLE suppression (“treatment”), and rabbit (“host”) on the abovementioned fitness measures were subsequently examined by full-factorial ANCOVA with “host” as a nested factor within “treatment.” Where “body size” had insignificant effect, it was excluded from the final analysis (weight conversion into eggs). Normal distribution and homogeneity of residuals was confirmed by Shapiro–Wilk and Bartlett’s tests, respectively. Hatching rates did not distribute normally or homogenously among treatment groups. In this case, differences were examined by the a-parametric Wilcoxon test after pooling the data of each “treatment” group. Proportions (weight conversion into eggs) were Logit-transformed. The effect of CLE on larval development was examined by one-way ANOVA.

(c) qPCR results: amplification data were analyzed as a relative standard curve experiment where the tick’s 18S rRNA gene served as the endogenous control, and DNA from a randomly chosen, 3-day-old, untreated female served as the calibrator sample (included in each plate). Briefly, 16S and 18S rRNA gene copy numbers were extrapolated using their corresponding standard curves, and relative quantities of the 16S gene were determined for each sample. Resultant data were normalized relative to the calibrator sample and log-transformed to homogenize variances among groups. Distributions were tested for normality (Shapiro–Wilk W test) and means were subsequently separated by Tukey HSD tests (*P* < 0.05). Saline injections had no effect on CLE densities in mature 40-day-old females, compared to untreated counterparts (ANOVA: *F* = 0.114, DF = 1, *P* = 0.743, *n* = 5 in each group, see also Figure 2 for a similar effect in teneral 3-day-old females), and these groups were pooled for subsequent analyses.

(d) Bacterial community analyses: Analyses were performed separately for each sequencing round (nymphs and adult ticks, respectively). Background noise was reduced by excluding rare OTUs appearing only once in the datasets (McCune et al., 2002). Arcsine-square-root transformed, relative abundance data were subsequently processed in PC-ORD v6.08 (MjM Software, United States). Multivariate analysis was performed using Bray–Curtis (Sorensen) index as a distance measure. Data were then ordinated using Principal Coordinate Analysis (PCoA) (Mather, 1976), and samples were clustered with flexible beta linkages (β = −0.25). Differences among groups were established by pairwise comparisons using the Multi-Response Permutation Procedure (MRPP; Mielke et al., 1976); α was Bonferroni-adjusted according to the number of comparisons, and the OTUs significantly differing between groups were detected by computing indicator values (IVs) as described by Dufrene and Legendre (1997).

Throughout the text, means are reported alongside their standard errors. Where covariate analyses were performed (nymph development and female fitness), least square means are reported.

## Results

Engorged nymphs of *R. sanguineus* ticks were injected either with antibiotics (aposymbiotic-CLE suppressed ticks) or with saline (symbiotic-control ticks), and the corresponding molted adults were later fed until repletion. CLE abundance, various fitness parameters, and the microbiome associated with nymphs and females were characterized and quantified in the different treatment groups during the tick life cycle (see experimental summary in Figure 1).

### CLE Abundance in Ticks and Their Suppression by Antibiotics

#### Nymphs and Teneral Adults

The occurrence of CLE in symbiotic ticks changed significantly throughout development from engorged nymphs to mature adults. Relative to host cells, CLE levels were low in 7-day post-repletion nymphs, elevated in teneral 3-day-old females, and further increased in mature females at 40 days post-eclosion (2.78- and 6.39-fold increase, respectively; Tukey HSD comparisons, *P* < 0.034; Figure 2A). Additionally, we observed that males (tested at 40 days post-eclosion) accommodated significantly fewer CLE than females of the same age group (3.86-fold lower abundance, Tukey HSD comparisons, *P* < 0.0024; Figure 2B) and in comparable densities to that of nymphs (Figures 2A,B). These results suggest that CLE remain dynamic during ontogeny and increase in number following ecdysis in females but not in males.

Contrary to symbiotic counterparts, 3-day-old females, previously treated with antibiotics as nymphs accommodated significantly reduced CLE levels regardless of the type of antibiotics used (rifampicin, tetracycline, or ofloxacin; Tukey HSD comparisons, *P* < 0.034; Figure 2A). Nevertheless, significant differences were noted in the potency of different antibiotics. Ofloxacin proved to be the most effective agent in eliminating CLE and reduced the density of the bacterium by 31.12 times compared to saline-injected counterparts. Similar doses of tetracycline or rifampicin were also effective, but to a lesser extent and reduced the occurrence of CLE in females by 3.67 and 6.62 times compared to saline or saline:DMSO-injected reciprocal controls (Figure 2A). Injections containing no antibiotics had negligible effects on CLE densities compared to symbiotic control ticks (treatments C2 and C3 compared to C1, respectively; Tukey HSD comparisons, *P* > 0.976, Figure 2A). When contrasted with CLE densities in nymphs, the effect size of all antibiotics was nonetheless smaller (11.37-, 1. 33-, and 2.98-fold decrease, OFX, TET, and RIF, respectively), suggesting that bacteria were suppressed mostly during the period to and following adult ecdysis.

#### Mature Unfed Adults

Due to its notable efficacy, ofloxacin was chosen for further testing the effects of the microbiome on tick development and reproduction. During these experiments, we found that the bactericidal effect of a single antibiotic dose given at the nymphal stage extended more than 60 days after treatment and remained valid (and further intensified) in 40-day post-eclosion females and males. These ticks continued to harbor minor amounts of CLE compared to saline-injected controls (134.56 and 23.65 times less, females and males, respectively; Tukey HSD comparisons, *P* < 0.0024; Figure 2B). Doubling the dose of ofloxacin to 100 ng/mg nymph weight did not further reduce CLE loads in females at 40 days post ecdysis [−2.14 ± 0.33 and −1.86 ± 0.06 Log relative abundance units, OFX and OFX(x2), respectively, Tukey HSD test, *P* = 0.085; Supplementary Figure S3], affirming the validity of our current antibiotic treatment protocol.

#### Fed Females

The effect of antibiotics remained significant in feeding females as well (Figure 2B). Initiation of feeding in symbiotic females was accompanied by an increase in body size (average weight: 8.396 ± 1.86 mg, compared to 3.004 ± 0.082 mg fed and non-fed females, respectively). Nevertheless, their CLE densities remained comparable to that of non-fed counterparts (Figure 2B), suggesting that symbionts multiply parallel to weight gain in females. Conversely, females injected with ofloxacin as nymphs, although having increased in weight (5.976 ± 1.71 mg, compared to 3.34 ± 0.075 mg; fed and non-fed females, respectively), harbored significantly less CLE than their symbiotic counterparts (206.004 times less; ANOVA, *F* = 162.98, DF = 1, *P* < 0.0001; Figure 2B). Comparable densities were recorded in a similar, preliminary experiment where CLE was found to be 269.05-fold more abundant in symbiotic fed females compared to ofloxacin-treated counterparts (ANOVA, *F* = 78.15, DF = 1, *P* < 0.0001, *n* = 5 in each group; results not shown). These results suggest that CLE is unable to recover after treatment with ofloxacin regardless of the apparently favorable conditions accommodated by feeding. This was supported by examining the density of CLE in females at the end of their oviposition. Here, CLE also remained suppressed in ticks formally treated with antibiotics and detected at significantly lower densities compared to untreated post-oviposition females (1812.48-fold less, ANOVA, *F* = 166.85, DF = 1, *P* < 0.0001; Figure 2B).

#### Eggs and Larvae

Diagnostic PCR using CLE-specific primers consistently detected the 16S rRNA of the bacterium in eggs and larvae of saline-injected females but not in corresponding counterparts from antibiotic-treated females (*n* = 18 eggs or larvae in each treatment group, Figure 2C). Simultaneously, detecting the tick’s 18S rRNA confirmed the presence of target DNA in all of these samples and validated our PCR procedures (Figure 2C). These trials demonstrated that the vertical transmission of CLE, normally achieved transovarially, was effectively reduced in antibiotic-treated females.

### Effect of CLE on Nymph Post-feeding Development

**FIGURE 3:**
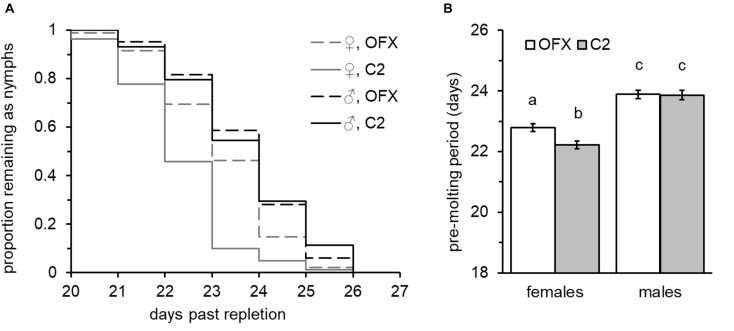
Effect of ofloxacin (OFX) on nymphal development: **(A)** Molting dynamics and **(B)** male and female nymph pre-molt period, following injections of saline (C2, shaded bars) or saline containing antibiotics (OFX, dosed at 50 ng/mg nymph weight, empty bars). Injections were performed 24–48 h after the nymphs had left their hosts. Different letters above means denote significant difference (Tukey HSD comparisons, *P* < 0.05; *n* = 44–95 in each group). Females developed faster than males and suffered a small but significant delay in molting following injection of antibiotics. Male development was not affected.

All nymphs successfully molted into viable adults following injections, suggesting that injury did not jeopardize the successful completion of development. Nevertheless, possibly due to the mild injury involved, injection had a small delaying effect on pre-molt period (Supplementary Figure S1), suggesting that resources were allocated to recovery. Nymph post-feeding development was positively correlated with nymph weight and significantly affected by sex in both treatment groups. Increased weight was significantly associated with longer development times in females and in all but one of the male groups (linear regression analysis, Supplementary Table S3). This was also evident when examined by full-factorial ANCOVA (overall “weight” effect: *F* = 80.85, DF = 1, *P* < 0.0001, no significant interaction with sex, treatment or replicate, Supplementary Table S4). Nevertheless, regardless of their weight and treatment group, females developed faster than males (ANCOVA, overall “sex” effect: *F* = 96.80, *P* < 0.0001; *post hoc* Tukey HSD comparisons, *P* < 0.0001, Figure 3), and on average molted into adults 22.5 ± 0.089 days after leaving their host – approximately 1.4 days earlier than male nymphs (mean development time: 23.87 ± 0.106 days). This effect was consistent among treatment groups and replicates (no significant interaction with treatment and replicate, Supplementary Table S4). Adding to the cumulative effects of weight and sex, “treatment” was an additional significant factor affecting development time. This was true for saline-injected female nymphs, which normally required 22.22 ± 0.128 days to complete their development and a further 0.57-day extension to 22.79 ± 0.124 days when injected with ofloxacin (ANCOVA followed by Tukey HSD tests, *P* = 0.009, Figure 3B). Nevertheless, males were not affected by treatment (ANCOVA, “treatment” × “sex” interaction: *F* = 3.89, *P* = 0.049) and both male treatment groups required similar time periods for completing their development and molting into adults (23.86 ± 0.162 and 23.88 ± 0.137 days, saline and ofloxacin-treated nymphs, respectively; ANCOVA followed by Tukey HSD tests, *P* = 0.999, Figure 3B). These sex-dependent treatment effects, however small, were consistently detected in both replicates (ANCOVA followed by contrast *t*-test comparisons for individual replicates, females: *t* > 2.12, *P* < 0.034; males: *t* < 0.39, *P* > 0.689; no significant interaction between treatment, sex and replicate, Supplementary Table S4). Similarly, survival analysis on pooled data from both replicates detected significant difference in molting period only for females (Wilcoxon test: χ^2^ = 22.08, *P* < 0.0001; χ^2^ = 0.009, *P* = 0.922, females: males, respectively), corroborating a sex-specific retarding effect of antibiotic treatment on nymph development.

Additionally, we found that doubling the dose of antibiotics to 100 ng/mg nymph weight did not further postpone nymph development time (replicated once, ANCOVA followed by contrast *t*-test comparisons, females: *t* = −0.48, *P* = 0.627, males: *t* = −1.47, *P* = 0.145; Supplementary Figure S3), suggesting that the effect of antibiotics on development was indirect and resulted consequently to suppression of bacteria.

### Effect of CLE on Female Fitness

#### Feeding Period

**FIGURE 4:**
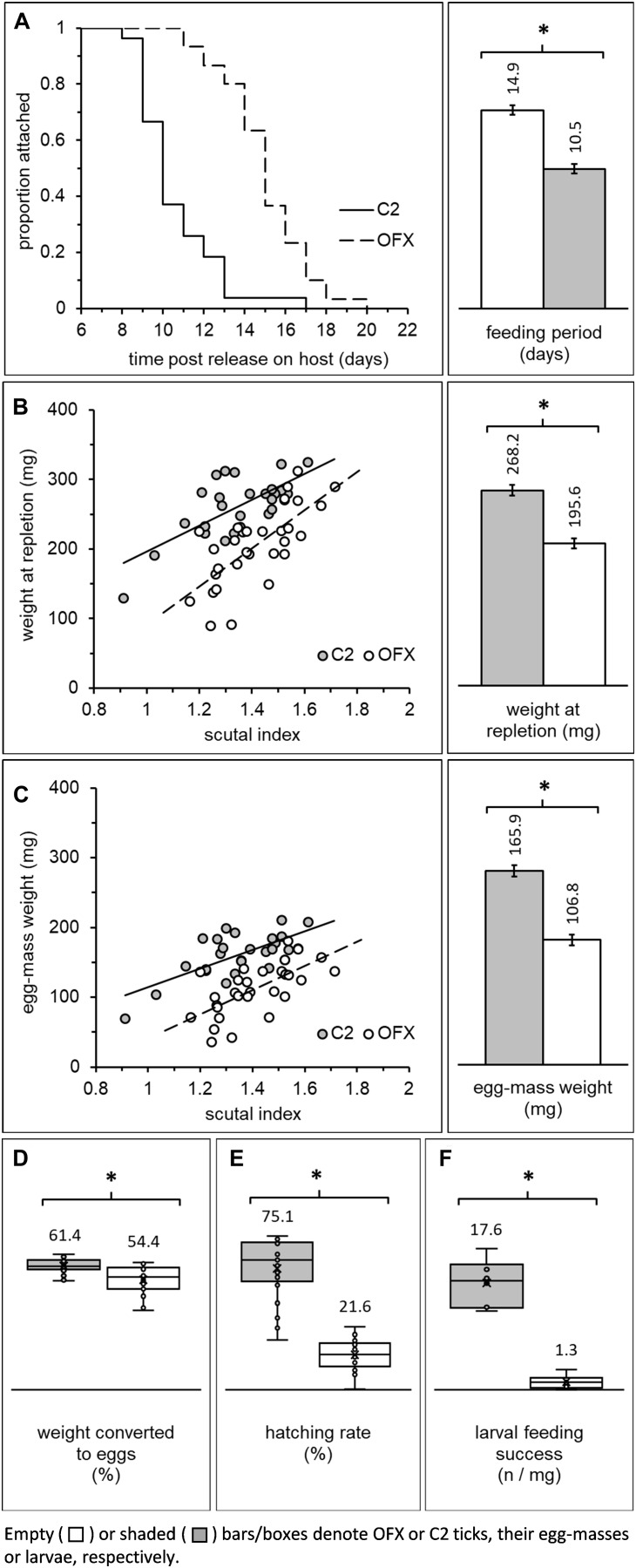
Fitness parameters of adult females previously injected as nymphs with ofloxacin (OFX) or saline (C2; empty and shaded bars/boxes, respectively): **(A)** Dynamics of detachment from the host and mean feeding period, **(B)** correlation between weight and body size (scutal index) and mean engorgement weight of replete females, **(C)** correlation between egg-mass weight and body size, and mean fecundity of females, **(D)** treatment-related differences in percent of weight converted to eggs and **(E)** egg hatching rate. Additionally depicted is **(F)** feeding success of larvae originating from antibiotic-treated and saline-injected females (number of replete larvae/mg larvae released on a host; see also Figure 2C depicting the detection of CLE in these larvae). Least square means or means (depicted by bar and box-plots, respectively) along with their standard errors are indicated in each figure. Asterisks (*) indicate significant differences among groups. Females: *n* = 27–30 in each group. Larvae: *n* = 6 replicates in each group.

Newly ecdysed females, having completed their pre-feeding period, mated and engorged on rabbits, and were subsequently used to determine the effect of CLE on feeding period, engorgement weight, fecundity (egg-mass weight) and egg hatching rate. Female feeding period continued for 8–20 days after release on the host, was not affected by body size in most groups (linear regression analysis, Supplementary Table S5, ANCOVA, “body size”: *F* = 3.94, DF = 1, *P* = 0.052, no significant interaction with “treatment”), and was significantly prolonged in aposymbiotic females (ANCOVA, “treatment”: *F* = 75.38, DF = 1, *P* < 0.0001; Figure 4A). On average, these females concluded feeding and left their hosts 14.86 ± 0.34 days post-release, approximately 4 days later than their symbiotic counterparts (10.46 ± 0.36 days until repletion). Some aposymbiotic females fed for substantial periods and detached while clearly not engorged (*n* = 7). Others fed to repletion but remained alive without producing any eggs (*n* = 2) or died soon after leaving their host (*n* = 1). The incidence of such events was rare in symbiotic females (*n* = 2), suggesting that suppressing CLE inflicted significant stress on feeding females. Indeed, all other fitness-related parameters that we quantified were negatively affected.

#### Engorgement Weight

Female engorgement weight was significantly and positively correlated with body size (scutal index) in both symbiotic and aposymbiotic ticks (linear regression analysis, Supplementary Table S5; ANCOVA, “body size”: *F* = 39.10, DF = 1, *P* < 0.0001, no significant interaction with “treatment,” Figure 4B). Nevertheless, regardless of their size and prolonged feeding period, aposymbiotic females concluded their feeding at significantly lower weight compared to symbiotic counterparts (ANCOVA, “treatment”: *F* = 55.73, DF = 1, *P* < 0.0001, Figure 4B). These females weighed approximately 73% of the final engorgement weight of symbiotic females (268.23 ± 7.05 and 195.55 ± 6.71 mg, C2 and OFX, respectively). Accordingly, the rate at which these females gained weight while feeding was 1.81 times lower than the average rate of symbiotic females (25.42 ± 6.06 and 14.01 ± 4.77 mg day^–1^, C2 and OFX, respectively). Thus, regardless of body size, suppression of CLE in female ticks was associated with longer feeding periods and reduced engorgement weight.

#### Fecundity (Egg-Mass Weight)

Similar to engorgement weight, egg-mass weight was significantly and positively correlated with body size in both treatment groups (linear regression analysis, Supplementary Table S5; ANCOVA: “body size”: *F* = 28.80, DF = 1, *P* < 0.0001, non-significant interaction with “treatment”). Regardless of their size and corresponding with their lower weight, aposymbiotic females produced significantly lighter egg-masses than their symbiotic counterparts (approximately 34% lighter, 165.92 ± 4.97 and 106.82 ± 4.73 mg, C2 and OFX, respectively; ANCOVA: “treatment”: *F* = 74.12, DF = 1, *P* < 0.0001, Figure 4C). Extrapolating the weight of single eggs (through weighing batches containing 69–226 eggs) demonstrated no treatment-related difference in the weight of individual eggs (ANOVA: *F* = 0.80, DF = 1, *P* = 0.394, 39.8 and 39.0 μg, C2 and OFX, respectively, *n* = 5 in each group), indicating that egg-masses of aposymbiotic females indeed contained fewer eggs.

Female weight conversion into eggs was not correlated with body size (linear regression, Supplementary Table S5). Females normally converted 61.36 ± 0.68% of their weight into eggs, but only 54.40 ± 1.22% when CLE were suppressed (ANOVA: “treatment” effect: *F* = 38.59, DF = 1, *P* < 0.0001, Figure 4D). Thus, the reduced fecundity of aposymbiotic females stems mainly from their lower engorgement weight but also from a reduced capacity to transform weight into eggs.

#### Fertility (Egg Viability)

Coupled with their reduced fecundity, aposymbiotic females suffered a decline in fitness due to severely reduced egg viability. Hatching rates were not correlated with body size regardless of treatment (linear regression, Supplementary Table S5) and averaged at 75.13 ± 3.78% in symbiotic egg-masses. Differently, eggs deposited by aposymbiotic females hatched at significantly lower rates (21.60 ± 1.82%, Wilcoxon test: χ^2^ = 35.04, DF = 1, *P* < 0.0001, *n* = 24–25 in each group, Figure 4E). Microscopic observations confirmed that embryos had developed within these eggs, apparently up to a very advanced stage, but were unable to hatch, and contrarily to symbiotic counterparts, accumulated excessive amounts of white deposits (possibly guanine) in their Malpighian tubules. Additionally, we noticed a unique hatching pattern in aposymbiotic egg-masses. Specifically, viable eggs that eventually hatched were distinctly clustered within the egg-mass and temporally were those that were last-produced by the females. Conversely, hatching occurred evenly in symbiotic egg-masses, where any dead eggs were randomly distributed.

### Effect of CLE on Larval Development

Larvae hatching from egg masses of symbiotic and aposymbiotic females were subsequently allowed to feed on a host, and engorged individuals were collected and counted. Comparing feeding success rates between treatment groups ([number of engorged larvae] × [mg larvae released on a host] ^–1^) showed that significantly fewer larvae completed their feeding when their progenitor females were treated with antibiotics (ANOVA: *F* = 98.81, DF = 1, *P* < 0.0001; 17.65 ± 1.57 and 1.29 ± 0.46 engorged larvae × mg larvae released^–1^, symbiotic and aposymbiotic females, respectively, Figure 4F). Additionally, while most engorged symbiotic larvae successfully molted into nymphs (84.84 ± 1.41%), none were able to do so when females were aposymbiotic. Corroborating these findings is the reduced CLE signal we found in these larvae (diagnostic PCR, see above).

### Characterization of Nymph and Adult Tick Microbiomes

Corresponding with our preliminary data (Supplementary Figure S4) and previous findings (Lalzar et al., 2012), we found the tick microbiome to be primarily composed of *Coxiella* sp. (CLE) bacteria (Supplementary Tables S6, S7). Other bacteria, excluding a few exceptions, usually contributed very small proportions of the detected community.

#### Nymph Microbiomes

**FIGURE 5:**
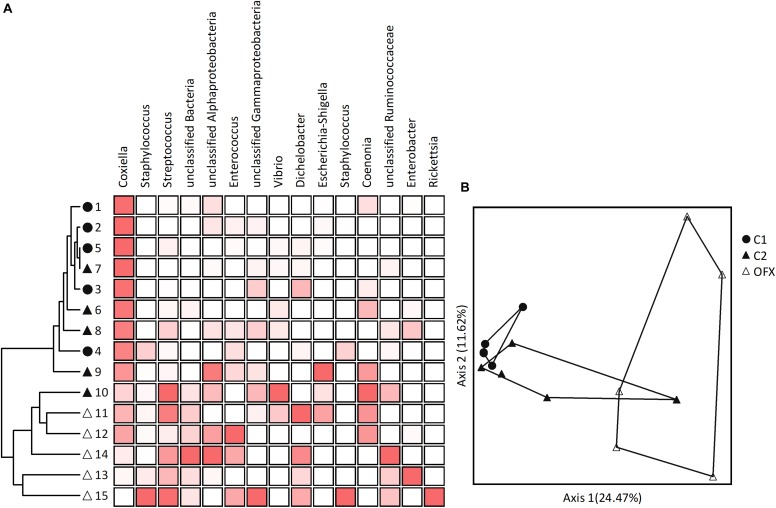
Microbiomes of nymphs: **(A)** Cluster and **(B)** Principal Coordinate Analyses based on Bray–Curtis distance measures, depicting similarities between microbiomes of untreated (C1, filled circles), saline-injected (C2, filled triangles), or antibiotic-treated (OFX, empty triangles) replete nymphs, sampled a week after injections (*n* = 5 in each group). Coupled to the cluster dendrogram is the abundance data matrix for the 15-most prevalent OTUs detected in our dataset. Color intensity is proportional to the relative abundance values in each column. Principal Coordinate scatterplots were ordinated according to the first two principal components representing the highest percentage of explained variability. Each point represents an individual sample. Prior to analyses, sequences were classified into OTUs having 97% sequence similarities (single and doubletons were excluded). A complete list of OTUs composing these microbiomes is available at Supplementary Table S6.

On average, CLE (OTU 1) contributed 96.71 ± 0.61 and 92.69 ± 2.48% of all reads in symbiotic, non-treated, and saline-injected nymphs, respectively. CLE remained dominant in ofloxacin-treated nymphs albeit to a lesser extent and comprised an average of 82.73 ± 2.51% of the remaining bacterial population (Figure 5A). Accompanying this core component of the nymph microbiome were 368 additional OTUs that were detected, each accounting for up to 7.99% of the reads in each sample (Supplementary Table S6). Consistently detected OTUs (appearing in all samples) other than CLE typically accounted for negligible proportions of the microbiome. These included *Staphylococcus* (0.91 ± 0.53%) and *Cutibacterium* (0.08 ± 0.02%) as well as multiple OTUs corresponding to *Coxiella*. Other bacteria were unevenly distributed among our samples. Overall, 101–155 bacterial genera assembled a typical nymph microbiome, the vast majority of which remained marginal compared to CLE (Supplementary Table S6). Accordingly, the alpha diversity in these samples (denoted by the Shannon and Simpson indices) remained small, particularly in non-treated and saline-injected nymphs (Supplementary Table S2).

Multi-response permutation tests (MRPP, adjusted α = 0.016) separated non-treated nymphs from their ofloxacin-treated counterparts (*A* = 0.098, *P* = 0.0018) but not from saline-injected nymphs (*A* = 0.034, *P* = 0.147). The latter group did not differ from ofloxacin-treated ticks (*A* = 0.038, *P* = 0.018). These patterns were confirmed by PCoA, which clustered microbiomes distinctly according to treatment (non-treated and ofloxacin-injected nymphs), or with some overlap (saline-injected with non-treated and ofloxacin-injected nymphs, Figure 5B). Indicator Species Analysis highlighted CLE (OTU 1 as well as other OTUs assigned as *Coxiella*) as accounting for the differences among non-treated and ofloxacin-treated nymphs (more abundant in non-treated ticks, IV = 51.4, *P* = 0.01). Other bacteria, which significantly differed among these microbiomes, were *Streptococcus* sp. unclassified bacteria 4 and 562, and *Portiera* – all of which occurred at very low numbers and were more abundant in ofloxacin-treated ticks, IV ≥ 61.8, *P* < 0.049; Supplementary Table S6). Differing between saline and ofloxacin-treated ticks were CLE (OTU 1 and other OTUs assigned as *Coxiella*; more abundant in saline-treated ticks; IV = 53.3, *P* = 0.033), and unclassified bacterium 4 as well as *Dichelobacter* (OTU 9), both occurring at low numbers and more abundant in ofloxacin-treated ticks (IV ≥ 74.1, *P* ≤ 0.049; Supplementary Table S6).

#### Female Microbiomes

**FIGURE 6:**
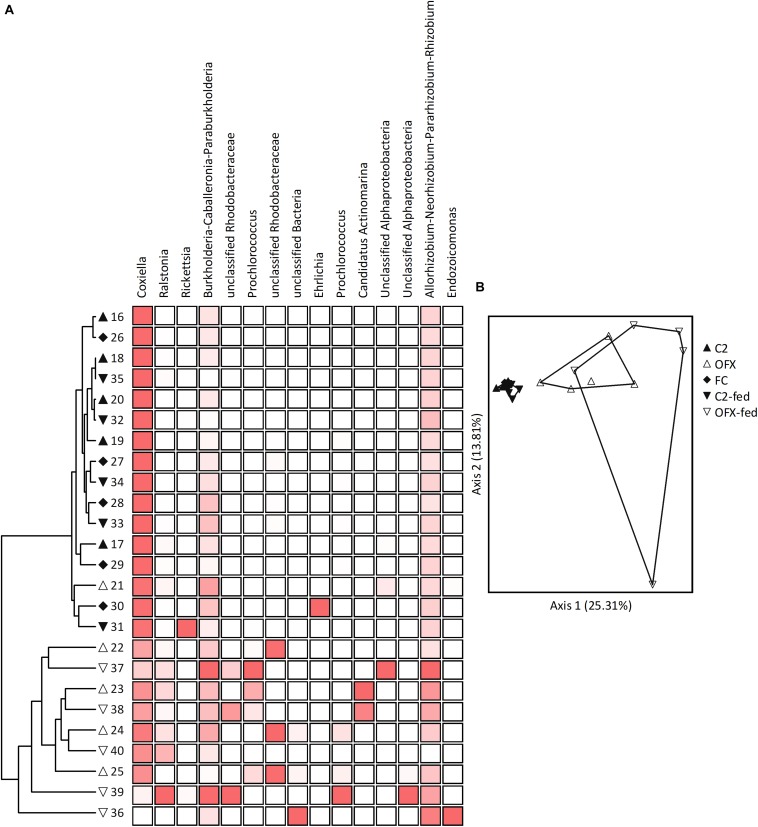
Microbiomes of females: **(A)** Cluster and **(B)** Principal Coordinate Analyses based on Bray–Curtis distance measures, depicting similarities between microbiomes of saline-injected, unfed (C2, filled triangles) or fed (C2-fed, inverted filled triangles) females, and antibiotic-treated, unfed (OFX, empty triangles), or fed (OFX-fed, inverted empty triangles) females (*n* = 5 in each group). Additionally depicted are field-collected, unfed females (FC, filled diamonds), *n* = 5. Coupled to the cluster dendrogram is the abundance data matrix for the 15 most prevalent OTUs detected. Color intensity is proportional to the relative abundance values in each column. Principal Coordinate scatterplots were ordinated according to the first two principal components representing the highest percentage of explained variability. Each point represents an individual sample. Prior to analyses, sequences were classified into OTUs having 97% sequence similarities (single and doubletons were excluded). See Supplementary Table S7 for a complete list of OTUs composing these microbiomes.

Adult females hosted a bacterial community similar in composition to the nymph microbiome. Clustering sequences across our dataset identified 498 OTUs, of which CLE (OTU 1) was by far the most dominant and consistently prevailed in all samples (Figure 6A). On average, sequences affiliated to CLE accounted for 99.77 ± 0.02 and 96.67 ± 0.1% of all reads in symbiotic females (saline-injected unfed and fed females, respectively) as well as in field-collected unfed females (99.69 ± 0.05%). Similarly, unfed and fed females treated with ofloxacin harbored a microbiome predominated by CLE (97.53 ± 0.6 and 93.18 ± 1.53% of obtained sequences, respectively; Figure 6A). An additional consistently detected OTU (*Burkholderia-Caballeronia-Paraburkholderia*) occurred at extremely low proportions and composed only a minor component of the microbiome across groups (pooled means: 0.03 ± 0.003%; Figure 6A). Various other bacteria, including OTUs corresponding to *Coxiella* occurred sporadically within microbiomes, mainly in antibiotic-treated ticks and were altogether rare (max proportion: 3.6%; Supplementary Table S7). Ultimately, the microbiomes of females were composed of 15–105 OTUs. Accordingly, diversity and evenness measures indicated a skewed community where reads were unevenly distributed among the taxa comprising each microbiome (Supplementary Table S2).

Pairwise comparisons among all female groups (MRPP tests, α = 0.005) revealed that microbiome composition remained consistent in saline-injected, unfed and fed females as well as in field-collected unfed females (*A* ≤ 0.006, *P* ≥ 0.152). Conversely, bacterial communities of ofloxacin-treated ticks, whether unfed or fed, differed significantly from microbiomes of symbiotic counterparts (*A* ≥ 0.081, *P* ≤ 0.004), but not from each other (*A* = 0.018, *P* = 0.081). Correspondingly, ordinating samples according to microbiome similarities by PCoA resulted in three distinct clusters: the tightly overlapping symbiotic female groups (unfed, fed, and field-collected females), the ofloxacin-treated unfed group, and the ofloxacin-treated, fed group (Figure 6B). Indicator Species Analysis revealed that CLE accounted for the main differences in the microbiome of symbiotic and ofloxacin-treated, unfed females (IV ≥ 50.6, *P* ≤ 0.009, more abundant in symbiotic females). One other OTU assigned as *Rhodanobacter* was significantly more abundant in symbiotic, field-collected ticks (IV = 79.3, *P* = 0.046) but nevertheless occurred at extremely low proportions (Supplementary Table S7). Following a similar pattern, CLE and four other OTUs were differently associated with symbiotic and ofloxacin-treated, fed females (IV ≥ 34.8, *P* ≤ 0.049). These were assigned as *Chitinophaga*, *Streptomyces*, an unclassified *Streptomycetaceae* and *Paenibacillus*, occurring at low abundance in both groups but found more frequently in symbiotic females (Supplementary Table S7). Except for CLE, and regardless of their contribution to the separation of groups, these bacteria had very low relative abundances in our samples (≤0.01% of the population).

## Discussion

Ticks (Acari: Ixodida) are considered monophyletic (Black et al., 1997), yet they developed independent, host-specific adaptations for blood-feeding, which correlate with hemostasis and immune responses of their hosts (Mans and Neitz, 2004). Ticks are additionally associated with several symbiont lineages that are closely related to known pathogenic bacteria, possibly acquired from the host (Duron et al., 2017). Thus, the host may also account for the diverse symbiont lineages of ticks. Presumably in all cases, and regardless of the associated bacteria, symbioses in ticks have converged to satisfy demands for essential nutrients missing in the diet.

In the current study, we illustrate the dependency of *R. sanguineus* on a CLE throughout ontogeny. We demonstrate that CLE predominates the microbiome of *R. sanguineus* nymphs and adult females. CLE remained dominant also in antibiotic-treated ticks regardless of their suppression. We show that injection of a single dose of antibiotics (ofloxacin) to engorged nymphs efficiently and dramatically reduced the bacterial load and CLE numbers in subsequent life-stages. By demonstrating the inability of CLE to recover over time, we support a direct effect of symbiont suppression (and not of the antibiotic), resulting in the fitness reduction we observed throughout the tick’s life cycle.

### CLE Loads Throughout Ontogeny

*Coxiella*-like endosymbiont loads in teneral symbiotic adult females were lower than that of mature (40-day-old) unfed counterparts. In symbiotic males, CLE levels were lower than that of females and comparable to that of repleted nymphs (Figure 2A). These results are consistent with previous findings (Lalzar et al., 2012) where CLE loads of field-collected *R. turanicus* (a member of *R. sanguineus* species group) were significantly lower in males compared to females and remained stable over much of the tick activity season in the field. These patterns suggest that CLE is more important in females compared to males; first, because in females CLE proliferates during early adult life, possibly in order to colonize the ovaries and be transmitted vertically to the next generation (Lalzar et al., 2014); and second, since during feeding, females consume much greater quantities of blood and have higher metabolic and physiological demands compared to males. Hence, CLE is expected to supplement missing nutrients at greater quantities in feeding females. We found that fed and unfed females harbor comparable CLE densities, suggesting that symbionts and host cells proliferate simultaneously with the increase in female weight during feeding. Unfortunately, we did not record the CLE densities in feeding males. However, males increase in weight only slightly during feeding, suggesting that their symbiont densities remain low compared to that of females.

### Nymph Performance

Post-feeding developmental period of nymphs is weight and sex dependent. Female nymphs developed faster than males, and for both sexes, heavier individuals took longer to complete development and molt into adults. Female nymphs injected with ofloxacin showed a delay in the pre-molt period. This delay was marginal but significant and was not observed in males or in nymphs treated with rifampicin or tetracycline (Supplementary Figure S1). Notably, in a previous study, tetracycline and rifampicin treatments of *A. americanum* nymphs resulted in reduced CLE loads, but had no effect on the pre-molt period (Zhang et al., 2017). We assume that suppression of CLE by antibiotics prolonged nymph development and contributed to the negative effect we recorded in subsequent life stages; however, the potency and timing of antibiotic treatment probably also affected the outcome of our bioassays. Specifically, we assume that extension of nymph development became measurable when symbionts were suppressed fast enough for a significant deficiency in a vital service to build up. This was most effectively achieved when nymphs were treated with ofloxacin, probably due to increased potency against CLE compared to other antibiotics. Accordingly, if CLE was suppressed earlier (e.g., in engorged larvae), any deficiencies compensated by symbionts and consequently nymph development may have been affected to a greater extent.

The sex-specific effect of symbiont suppression and the accelerated development of female nymphs compared to males could stem from their initial difference in CLE loads. Thus, the increase in CLE populations observed in teneral females may have been initiated during the late stages of nymph development and accounted for their faster development. Correspondingly, female nymphs may be more susceptible and respond more acutely to reduction in CLE loads than males.

### Adult Fitness

The subsequent effect of CLE suppression on adult females is first evident by their extended feeding period. Following attachment, the rate of weight gain was reduced nearly twofold in aposymbiotic females, which subsequently prolonged their overall feeding time. The engorgement weight of these females was significantly reduced and was processed less efficiently into eggs, ultimately leading to their lower fecundity. These results suggest that CLE is required mostly during on-host feeding and processing of the blood meal, and to a lesser extent during oogenesis (occurs during the last step of feeding). Similarly, in another study, egg production was reduced when *R. microplus* females fed on a host treated with antibiotics but not when antibiotics were administered directly to engorged females (Guizzo et al., 2017). Similar results were recorded in *A. americanum* and *H. longicornis* females treated with antibiotics as engorged adults (Zhong et al., 2007; Zhang et al., 2017; Li et al., 2018). These studies, together with our results, support the idea that CLE is active mostly during feeding, and highlight the need to precisely time antibiotic treatments in order to accurately determine the contribution of symbionts to the fitness of ticks.

### Eggs and Larvae

Eggs of aposymbiotic females contain undetectable levels of CLE (diagnostic PCR, Figure 2C). Thus, although CLE was present in adults that molted from antibiotic-treated nymphs (determined by qPCR and high-throughput sequencing), it was probably not transmitted further to developing oocytes as would normally occur (Lalzar et al., 2014). Accordingly, CLE transmission is probably not essential for oocyte maturation process within the ovaries. It is possible that the remaining CLE (presumably surviving in the Malpighian tubules) are dedicated to support the basic physiology of the female tick, as is the case with low quantity of CLE in normal males (Lalzar et al., 2012).

Typically, symbiotic egg-masses contained few non-viable eggs, which were randomly distributed within the egg-mass. However, we observed that aposymbiotic egg-masses are composed of a major cluster of non-viable eggs and a minor, distinct portion of viable eggs produced at the end of the oviposition period. Larvae that hatched from such eggs were also aposymbiotic, suggesting that CLE does not directly affect these hatching patterns. This hatching pattern may be explained in view of the terminal investment hypothesis (Williams, 1966; Clutton-Brock, 1984), which predicts higher investment in offspring production as the parent ages. Although hard ticks are semelparous [producing offspring only once, but see Fisher and Blomberg (2011)], we postulate that the later laid eggs could complete their development since the female, which will die after finishing to oviposit, is able to invest any remaining nutrients scavenged from her body in the last developing oocytes. This practice may imitate the nutrient contribution of CLE to the symbiotic developing embryo (e.g., vitamins and co-factors) from tissue breakdown. Finally, we demonstrate that aposymbiotic larvae also showed difficulties in attachment to the host and were unable to complete their feeding, ultimately pointing that CLE is mandatory during feeding and processing of the blood meal.

### Microbiome

As expected and demonstrated in other studies (Lalzar et al., 2012; René-Martellet et al., 2017), the microbial community of *R. sanguineus* ticks used in the study was dominated by CLE, regardless of treatment or developmental stage. Nevertheless, bacterial community structure was different between symbiotic and aposymbiotic nymphs and adults. In both cases, reduced CLE levels in antibiotic-treated ticks contributed significantly to these differences. Other bacteria that significantly differed among ofloxacin-treated and non-treated nymphs and females were generally rare and accounted for extremely low proportions of the bacterial community associated with the ticks. These probably include minor populations internally associated with the ticks (e.g., in gut and salivary glands), but also reminiscent bacteria adhering to the exterior tick body which were not eliminated after surface sterilization. Although we cannot rule out a possible effect of such bacteria on fitness, their low abundance compared to CLE questions this possibility. Rare bacteria detected in the samples may differ in prevalence compared to previous findings, either because each study used different 16S rRNA gene region for sequencing, different analysis methodology, and/or different origin of the ticks (lab culture vs. field collected ticks). The contribution of such less prevalent bacteria to the observed results will require a further detailed study.

### Summary and Conclusion

Few studies examined the fitness consequences associated with elimination of endosymbionts in hard ticks (Zhong et al., 2007; Guizzo et al., 2017; Zhang et al., 2017; Li et al., 2018). Each study used either a different antibiotic or administration method and measured quantity of bacteria and fitness traits in somewhat different ways. Thus, although a similar trend of the essential contribution of CLE to the well-being of its host is consistent across those studies, it is difficult to compare them to our study. We used the bactericidal ofloxacin to suppress CLE, while other studies used rifampicin or tetracycline (bactericidal and bacteriostatic, respectively) which we found to be less effective in suppressing CLE. Interestingly, genomic evidence suggests that CLE from *R. sanguineus* may carry native resistance genes to tetracycline [by involvement of translation elongation factor G (Taylor and Chau, 1996; Tsementzi et al., 2018)], which could explain its relatively mild effect on CLE observed in our study. One re-occurring concern is a possible direct effect of the antibiotic contributing to the observed negative outcomes on fitness (see Li et al., 2018). We could not find a dose-dependent effect of ofloxacin on development time of nymphs, suggesting a negligible direct effect on the ticks. Additionally, our study is unique in that it measured fitness indices on adults and larvae that were not treated directly with antibiotics (by injection or feeding) and accordingly details the exact reproductive loss (in terms of weight gain, fecundity, egg viability, and larval competence) attributed to elimination of symbionts.

A general consistency among endosymbionts of obligate blood-feeders is their tendency to retain their B vitamins and co-factor synthesis pathways regardless of massive genomic erosion (Manzano-Marín et al., 2015). These micronutrients are suggested to be produced for the symbiont themselves (Serbus et al., 2017) and for their hosts (e.g., Hosokawa et al., 2010; Duron et al., 2018). CLE genomes differ considerably in completeness of B vitamin synthesis pathways (Tsementzi et al., 2018), and nutritional complementation evidence is not yet available for hard ticks. Thus, additional putative nutritional supplements of CLE to their tick hosts are suggested. These could include amino acids such as L-proline (Michalkova et al., 2014; Tsementzi et al., 2018), which may also be involved in the blood digestion process, energy production, and nitrogen metabolism as in other arthropods (O’Donnell and Donini, 2017). Further genomic and nutritional assays are needed to elucidate the exact contribution of CLE to the nutrition of their hard tick hosts.

Our study strengthens the hypothesis that CLE contribution to its *R. sanguineus* host is mostly required under high physiological demands such as blood meal processing and egg production, presumably via supplementing the host with essential micronutrients (such as B vitamins) and/or macronutrients (such as amino acids). The exact nature of this contribution is nevertheless still difficult to pinpoint. While this may apply primarily to females, males may also suffer fitness consequences from loss of their association with CLE. Complementation experiments are needed to further examine these hypotheses.

## Data Availability Statement

The datasets generated in this study have been deposited into the Sequence Read Archive (https://www.ebi.ac.uk/ accession: ERP118745, Samples: ERS4134016 – ERS4134055).

## Ethics Statement

The animal study was reviewed and approved by the ethic committee of the Kimron Veterinary Institute (approval # 020_B11136_16), and by the ethic committee of the Hebrew University Authority for biological and biomedical models (# MD-17-14900).

## Author Contributions

YG, MB-Y, and AR contributed to the conception and design of the study. MB-Y, AR, MM, EK, and AB performed the feeding experiments and molecular analyses. MB-Y performed the statistical analysis. YG and MB-Y wrote the first draft of the manuscript. All authors contributed to the manuscript revision, and read and approved the submitted version.

## Conflict of Interest

The authors declare that the research was conducted in the absence of any commercial or financial relationships that could be construed as a potential conflict of interest.

## Footnotes

1 http://bioinfo.ut.ee/primer3-0.4.0/

2 http://www.mothur.org/wiki/MiSeq_SOP
