# Supplementary material for: Coxiella-Like Endosymbiont of Rhipicephalus sanguineus Is Required for Physiological Processes During Ontogeny
Source: Front Microbiol. 2020 Apr 22;11:493. doi: 10.3389/fmicb.2020.00493 (PMC7188774; doi:10.3389/fmicb.2020.00493)
Supplement: Supplementary file 1 [file Data_Sheet_1.docx]

Supplementary Material

# Supplementary Data

**Preliminary analysis of the effect of antibiotics and injury by injection on nymph pre-molt period:** coupled to estimation of antibiotic efficiency in suppressing CLE we did a preliminary survey using a limited number of ticks to assess the effect of different antibiotics on nymph development. Assays proceeded as described in the main text. Means were separated for males and females by analysis of co-variance (ANCOVA) with 'treatment' as a fixed factor and 'nymph weight' as a co-variate. These experiments suggested that ofloxacin prolonged development in females but not in males. The effects of other antibiotics were less clear and suggested a shortening of development by DMSO or rifampicin or no-effect by tetracycline (ANCOVA followed by Tukey HSD tests, figure S1). These results together with antibiotic efficiency assays (main text, figure 2A) highlighted ofloxacin as a good candidate antibiotic – having potent effect on CLE but negligible direct effects on the ticks. Further experiments with a larger sample size showed that injury (saline injection, C2) had a slight but significant retarding effect on female nymph pre-molt period compared to non-treated controls (C1). Development time was further significantly prolonged by ofloxacin. These effects were not apparent in males (result no shown). Consequently, data analysis tested the effects of antibiotics, and nymph weight and sex on development time of antibiotic and saline-injected counterparts.


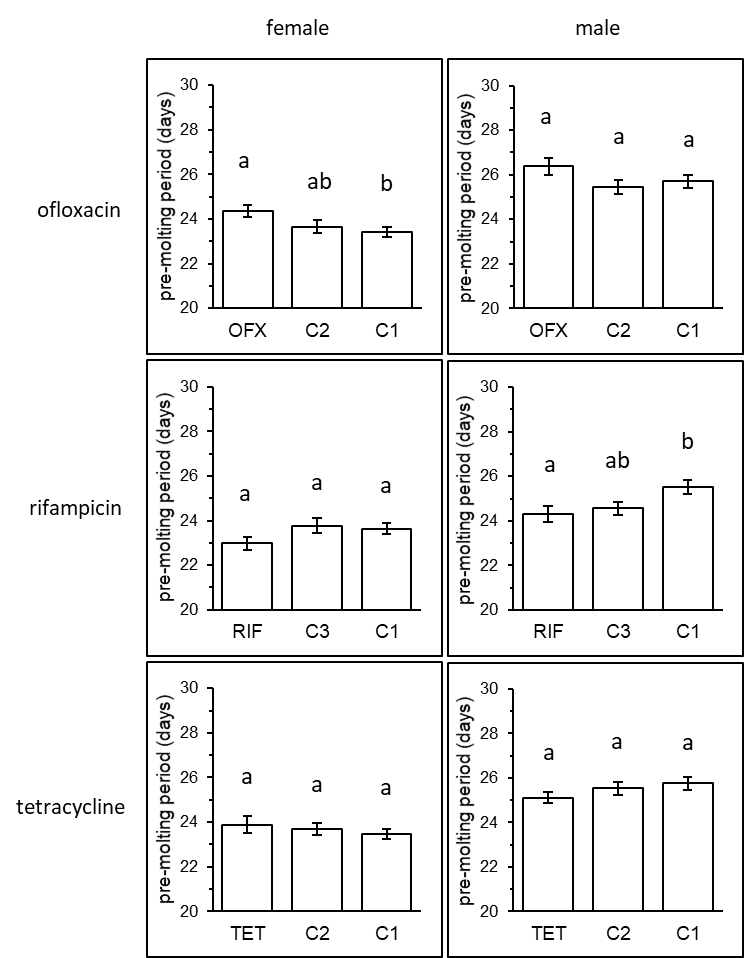


**Figure S1:** pre-molt development time of female and male nymphs injected with ofloxacin, rifampicin or tetracycline (ANCOVA followed by Tukey HSD comparisons). Treatment with ofloxacin (OFX) significantly prolonged female nymph development compared to non-treated controls (C1). Contrast t-test comparison with saline-injected counterparts (C2) was nevertheless, marginally significant (t = 1.78, P = 0.081). Treatments with rifampicin (RIF) or its reciprocal control (DMSO in saline, C3) were associated with a shorter pre-molt period in males. Tetracycline (TET) had no effect on pre-molt period. Different letters above means denote significant difference (n = 7 - 21 in each group, α = 0.05).


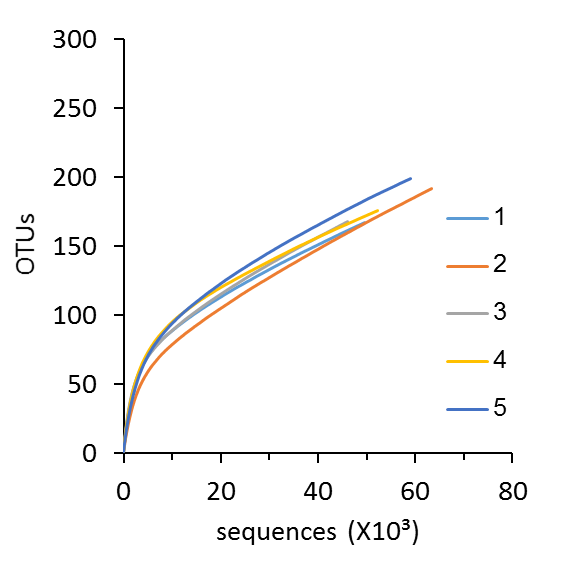

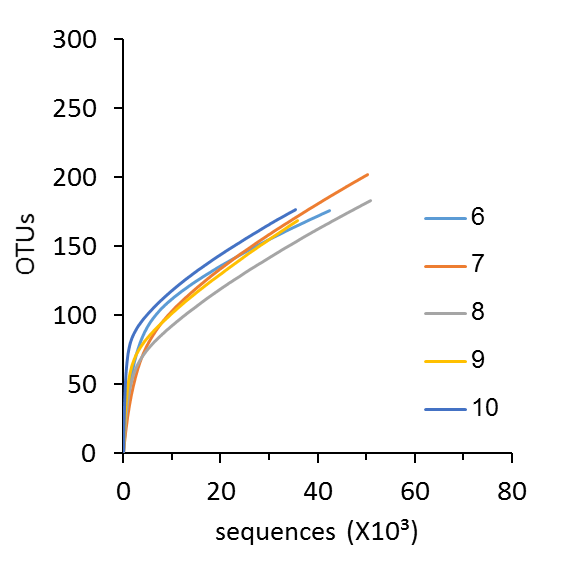

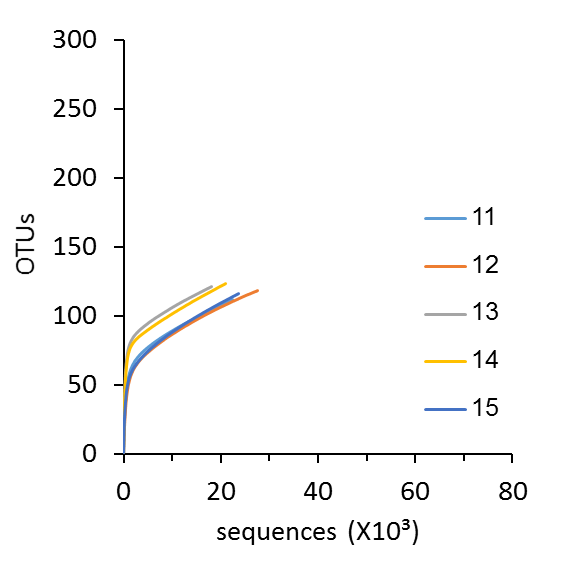

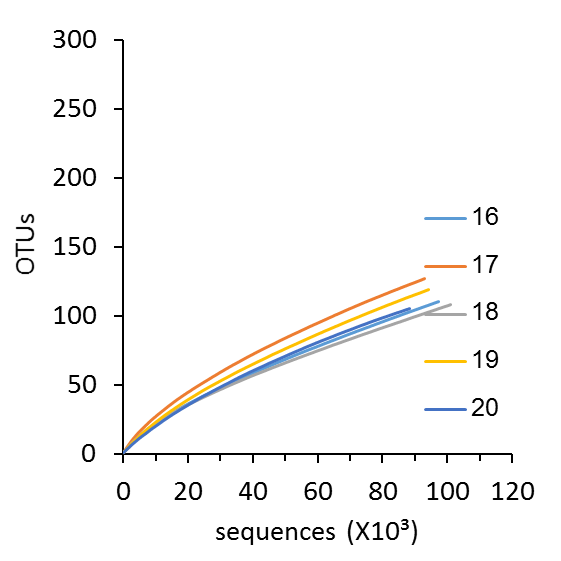

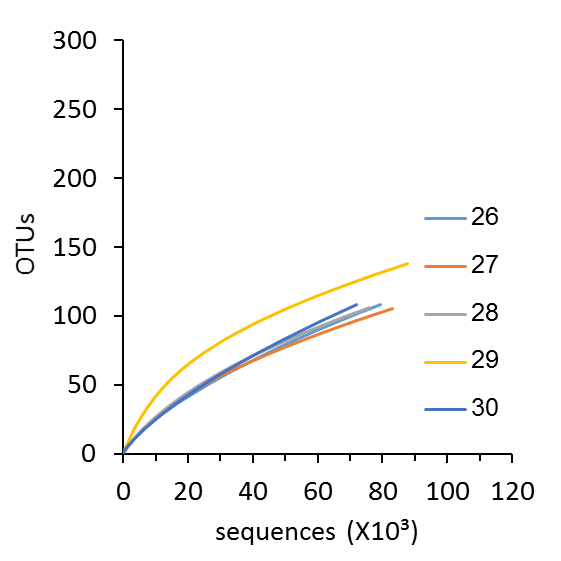

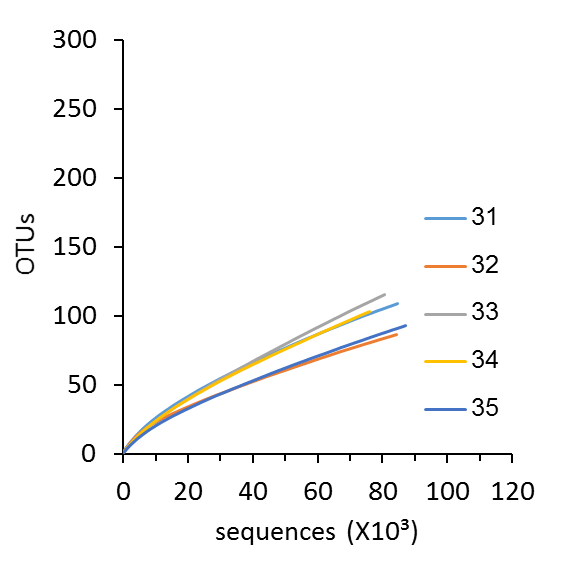

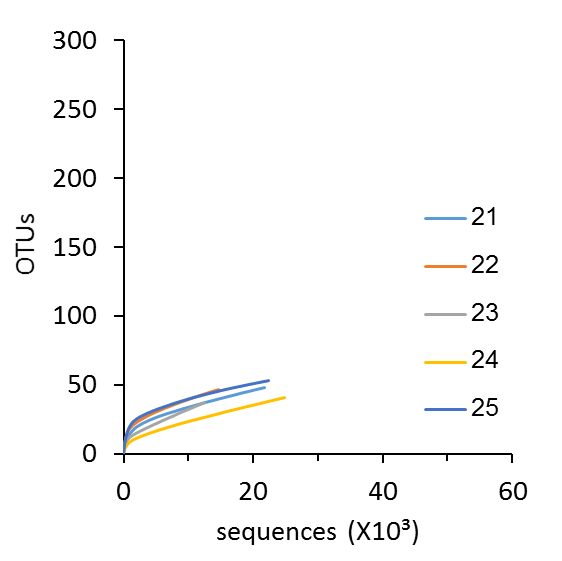

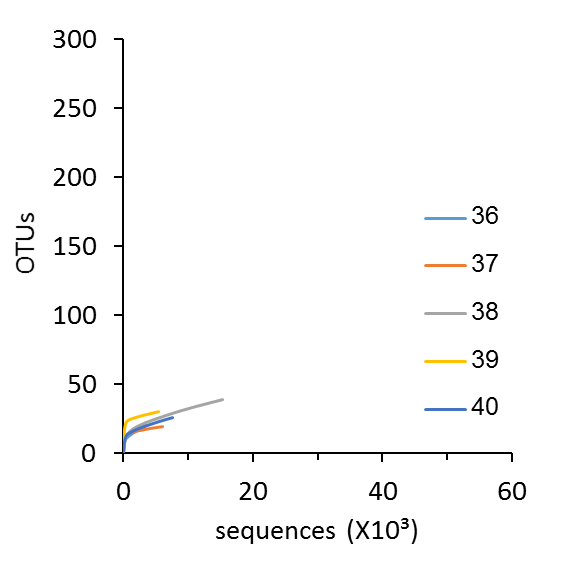


a

b

c

d

e

f

g

h

**Figure S2:** rarefaction curves of raw sequencing data depicting OTU abundance as a function of sampling effort in (a-c) nymphs (a – untreated, C1; b – injected with saline, C2; or c – injected with ofloxacin, OFX), and (d-h) adult females (d – unfed, injected with saline, C2; e – unfed, field-collected, FC; f – fed, injected with saline, C2-fed; g – unfed, injected with ofloxacin, OFX; h – fed, injected with ofloxacin, OFX-fed). Each colored line represents a single sample (#1-40).


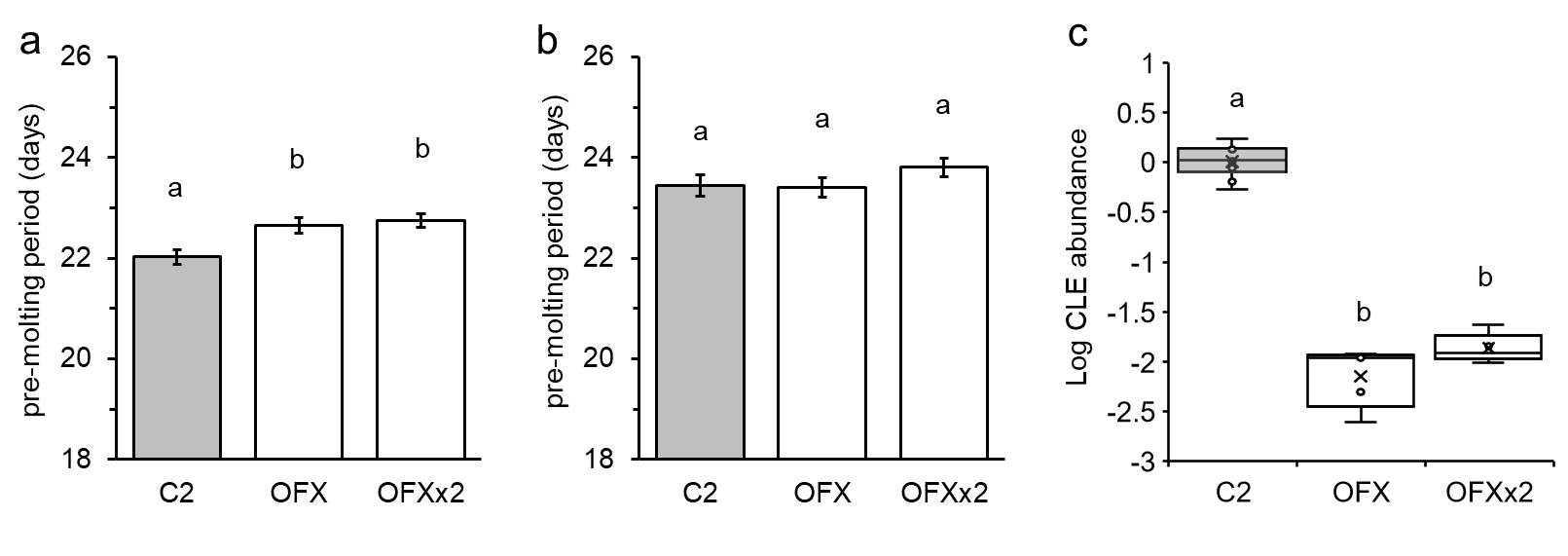
**Effect of doubling the dose of ofloxacin on nymph development time and adult CLE titer:** as reported in the main text females but not males had a significantly prolonged pre-molt period following treatment with ofloxacin (50ng / mg nymph body weight). Doubling the dose of ofloxacin to 100ng/mg nymph weight did not further prolong nymph development (ANCOVA followed by contrast t-test comparisons, females: t = -0.48, P = 0.627, males: t = -1.47, P = 0.145, or Tukey HSD tests, figure S2a and b). Additionally CLE loads in females at 40-days post ecdysis were significantly reduced in ticks injected with ofloxacin and were not affected by a double dose of antibiotics (-2.14 ± 0.33 and -1.86 ± 0.06 Log relative abundance units, OFX and OFX(x2) respectively, Tukey HSD test, P = 0.085, n=5 in each group, figure S2c), These results suggest that suppression of CLE was effectively achieved by a low dose of ofloxacin and that the effect of antibiotics on nymph development was indirect and resulted consequently to suppression of bacteria.

**Figure S3:** pre-molt development time of female **(a)** and male **(b)** nymphs, and relative abundance values of CLE in adult 40-day old female ticks **(c)** injected as nymphs with saline (C2), saline containing ofloxacin dosed at 50ng / mg body weight (OFX) or 100 ng / mg body weight (OFXx2). For both males and female nymphs doubling the antibiotic dose had no adverse effect on development time (ANCOVA followed by Tukey HSD comparisons, n = 22 - 37 in each group). Similarly, the relative abundance of CLE was not affected by a double dose of ofloxacin (Tukey HSD comparisons, n = 5 – 10 in each group). Different letters above means denote significant difference (α = 0.05).

**Preliminary survey of the bacterial community associated with untreated and antibiotic-treated nymphs:** this survey was carried out using a similar protocol described in the main text with a few exceptions. DNA was extracted from untreated and ofloxacin, rifampicin and tetracycline-injected nymphs, 7 days post-injections (n = 5 in each group). Sequencing was performed using the 341F-806R primer pair targeting the V3 and V4 regions of the bacterial 16S rRNA gene. Obtained sequences were processed and analyzed according to the standard MiSeq protocol (<http://www.mothur.org/wiki/MiSeq_SOP>) and phylotyped into operational taxonomic units (OTUs) corresponding to the genus level. Single and doubletons as well as rare genera occurring only once in the dataset were removed, and the relative abundance of the resulting OTUs was subsequently examined.

**
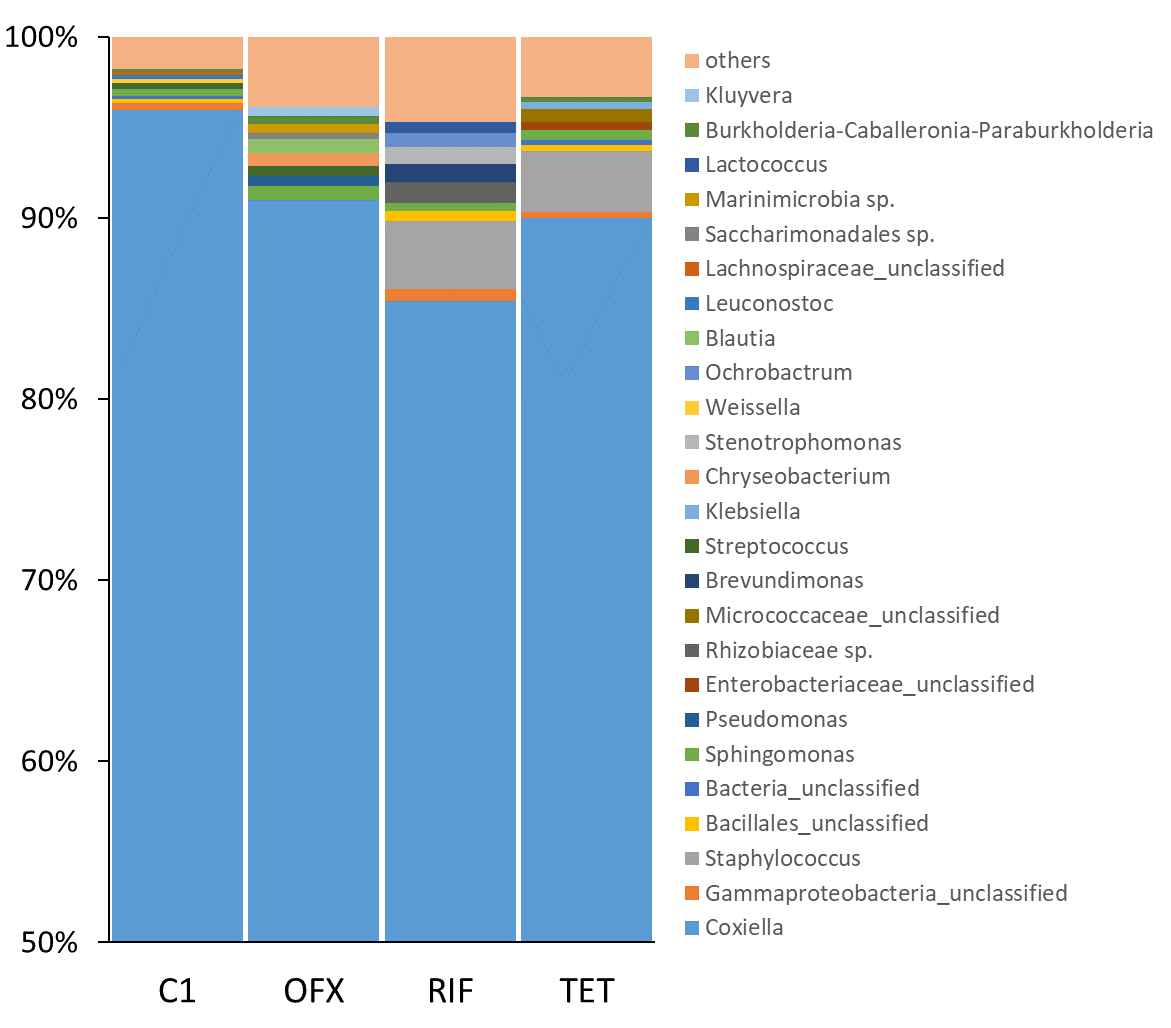
**

**Figure S4:** Composition of the bacterial community associated with replete, untreated nymphs (C1) and counterparts injected with ofloxacin (OFX), rifampicin (RIF) or tetracycline (TET). Nymphs were sampled and processed 7 days post-injections (n = 5 in each group). Average relative abundance values of the 10 most abundant genera detected in each treatment group are depicted. Other bacteria having lower abundance were grouped as 'others'. *Coxiella* was the predominant bacterial taxon associated with the nymphs of all treatment groups and remained notably abundant in C1 nymphs (95.96 ± 0.92%). Antibiotic-treated nymphs and particularly RIF-treated nymphs had reduced levels of *Coxiella* (91 ± 3.4%, 89.99 ± 4.11% and 85.43 ± 9.32%; OFX, TET and RIF, respectively). Rifampicin and tetracycline-treated nymphs additionally contained elevated proportions of *Staphylococcus* bacteria (3.72 ± 3.61% and 3.37 ± 2.9%; respectively). These trends emanated mostly from single individuals having 14.95 – 18.19% of their obtained sequenced identified as *Staphylococcus.*

| **target organism** | **gene** | **primer** | **sequence** | **amplicon size (bp)** | **qPCR^1^ / diagnostic PCR^2^ conditions**  **(x40 / x30 cycles, respectively)** | | **standard curve specifications** | | | **reference** |
| --- | --- | --- | --- | --- | --- | --- | --- | --- | --- | --- |
|  |  |  |  |  |  |  | **(mean ± SD, n = 12)** | | |  |
|  |  |  |  |  |  |  | **Slope** | **R2** | **Efficiency** |  |
|  |  |  |  |  |  |  |  |  |  |  |
| Coxiella | 16S rRNA | Cox 434f | 5’-CCTTTTGAGCGTTGACGTTA-3’ | 565 | initial denaturation | 95⁰c, 15^1^ / 5^2^ min | -3.52 ± 0.009 | 0.99 ± 0.001 | 91.99 ± 0.32 | Lalzar *et al.* 2012 |
|  | 16S rRNA | Cox 1004r | 5’-CCAAAGGCACCAAGTCATTT-3’ |  | denaturation | 95⁰c, 30 sec |  |  |  |  |
|  |  |  |  |  | annealing | 60⁰c, 30 sec |  |  |  |  |
| Ixodidae | 18S rRNA | Rh 673f | 5’-CTGAACATCATGCCGGTTCTTTC-3’ | 239 | extension | 72⁰c, 30 sec | -3.41 ± 0.009 | 0.99 ± 0.001 | 96.18 ± 0.37 | this study |
|  | 18S rRNA | Rh 909r | 5’-GGTCCAAGAATTTCACCTCTAGC-3’ |  | final extension | melting protocol^1^ 72⁰c, 5 min^2^ |  |  |  |  |
|  |  |  |  |  |  |  |  |  |  |  |

**Table S1:** primers used for detection and quantification of CLE by quantitative and diagnostic PCR. Reaction conditions remained similar for both procedures except for initial denaturation (15 or 5 min, respectively), number of cycles (x40 / x30 cycles, respectively), and the final extension stage (melting protocol or 5 min at 72°C, respectively).

**Table S2:** sequencing data and diversity indices of the bacterial microbiome associated with untreated (C1), saline-injected (C2) and antibiotic-injected (OFX) nymphs and females as well as field collected (FC) females. Sequencing was performed using the 515F-806R primer pair on an Illumina MiSeq machine. Sequences were classified into Operational Taxonomic Units (OTUs) differing by 3% sequence dissimilarity according to Mothur's online protocol. Further processing to remove rarely occurring OTUs was performed in PC-ORD.

**Table S3:** linear regression of post-feeding development time by weight in saline-injected (C2) and antibiotic-injected (OFX) nymphs. Development time was positively and significantly associated with nymph weight in both males and females.

|  | | | | | | | |
| --- | --- | --- | --- | --- | --- | --- | --- |
|  | **Rep.** | **n** | **Treatment** | **Regression** | | |  |
|  |  |  |  |  |  |  |  |
|  |  |  |  | **t** | **P** | **R^2^** |  |
|  |  |  |  |  |  |  |  |
|  | **females** | | | | | |  |
|  | 1 | 33 | OFX | 3.14 | 0.0037* | 0.241 |  |
|  |  | 36 | C2 | 2.81 | 0.0082* | 0.188 |  |
|  | 2 | 62 | OFX | 5.12 | <0.0001* | 0.303 |  |
|  |  | 45 | C2 | 5.31 | <0.0001* | 0.396 |  |
|  |  |  |  |  |  |  |  |
|  | **Males** | | | | | |  |
|  | 1 | 22 | OFX | 1.93 | 0.068 | 0.156 |  |
|  |  | 19 | C2 | 2.92 | 0.0095* | 0.334 |  |
|  | 2 | 60 | OFX | 4.96 | <0.0001* | 0.298 |  |
|  |  | 25 | C2 | 3.82 | 0.0009* | 0.387 |  |
|  |  |  |  |  |  |  |  |

**Table S4:** full-factorial ANCOVA determining the effects of treatment (saline vs. antibiotic injections), weight, sex and replicate on nymph post-feeding development time.

|  | | | | | |
| --- | --- | --- | --- | --- | --- |
| **Factor** | | **DF** | **F Ratio** | **Prob > F** | |
|  |  |  |  |  |  |
|  | Rep. | 1 | 28.0042 | <.0001* |  |
|  | Treatment | 1 | 4.5132 | 0.0345* |  |
|  | Rep.*Treatment | 1 | 0.0231 | 0.8792 |  |
|  | nymph weight (mg) | 1 | 80.8504 | <.0001* |  |
|  | Rep.*nymph weight (mg) | 1 | 1.0164 | 0.3142 |  |
|  | Treatment*nymph weight (mg) | 1 | 0.2407 | 0.6241 |  |
|  | Rep.*Treatment*nymph weight (mg) | 1 | 0.1005 | 0.7515 |  |
|  | Sex | 1 | 96.8037 | <.0001* |  |
|  | Rep.*Sex | 1 | 0.4476 | 0.504 |  |
|  | Treatment*Sex | 1 | 3.8955 | 0.0494* |  |
|  | Rep.*Treatment*Sex | 1 | 0.2182 | 0.6408 |  |
|  | nymph weight (mg)*Sex | 1 | 0.5423 | 0.4621 |  |
|  | Rep.*nymph weight (mg)*Sex | 1 | 0.4129 | 0.521 |  |
|  | Treatment*nymph weight (mg)*Sex | 1 | 0.0235 | 0.8784 |  |
|  | Rep.*Treatment*nymph weight (mg)*Sex | 1 | 0.1906 | 0.6628 |  |
|  |  |  |  |  |  |

**Table S5:** linear regression of female feeding period, engorgement weight, fecundity, weight conversion ratio and hatching rate by body size. † : n = 14 (host 1), 12 (host 2), 11 (host 3) ,12 (host 4).

|  | | | | | | | |
| --- | --- | --- | --- | --- | --- | --- | --- |
|  | **Host** | **n** | **Factor** | **Regression** | | |  |
|  |  |  |  |  |  |  |  |
|  |  |  |  | **t** | **P** | **R^2^** |  |
|  |  |  |  |  |  |  |  |
|  | **Females injected as nymphs with saline (C2-fed)** | | | | | |  |
|  | 1 | 14 | feeding period | 1.01 | 0.331 | 0.078 |  |
|  |  |  | weight | 2.86 | 0.0143* | 0.405 |  |
|  |  |  | fecundity | 2.31 | 0.039* | 0.307 |  |
|  |  |  | weight / eggs ratio | 0.65 | 0.524 | 0.034 |  |
|  |  |  | hatching rate† | 1.67 | 0.12 | 0.189 |  |
|  |  |  |  |  |  |  |  |
|  |  |  |  |  |  |  |  |
|  |  |  | feeding period | -3.1 | 0.01* | 0.465 |  |
|  | 3 | 13 | weight | 5.69 | 0.0001* | 0.746 |  |
|  |  |  | fecundity | 4.7 | 0.0006* | 0.668 |  |
|  |  |  | weight / eggs ratio | 1.67 | 0.122 | 0.202 |  |
|  |  |  | hatching rate† | -0.24 | 0.816 | 0.006 |  |
|  |  |  |  |  |  |  |  |
|  | **Females injected as nymphs with ofloxacin (OFX-fed)** | | | | | |  |
|  | 2 | 12 | feeding period | -0.33 | 0.745 | 0.011 |  |
|  |  |  | weight | 3.26 | 0.0085* | 0.515 |  |
|  |  |  | fecundity | 3.32 | 0.0078* | 0.523 |  |
|  |  |  | weight / eggs ratio | 1.07 | 0.309 | 0.102 |  |
|  |  |  | hatching rate† | 0.11 | 0.911 | 0.001 |  |
|  |  |  |  |  |  |  |  |
|  |  |  |  |  |  |  |  |
|  | 4 | 18 | feeding period | -0.61 | 0.549 | 0.022 |  |
|  |  |  | weight | 2.74 | 0.014* | 0.319 |  |
|  |  |  | fecundity | 1.86 | 0.082 | 0.177 |  |
|  |  |  | weight / eggs ratio | -0.59 | 0.565 | 0.021 |  |
|  |  |  | hatching rate † | -0.45 | 0.659 | 0.02 |  |
|  |  |  |  |  |  |  |  |
